# Lysophosphatidate Induces Chemo-Resistance by Releasing Breast Cancer Cells from Taxol-Induced Mitotic Arrest

**DOI:** 10.1371/journal.pone.0020608

**Published:** 2011-05-27

**Authors:** Nasser Samadi, Raie T. Bekele, Ing Swie Goping, Luis M. Schang, David N. Brindley

**Affiliations:** Department of Biochemistry (Signal Transduction Research Group), School of Molecular and Systems Medicine, University of Alberta, Edmonton, Alberta, Canada; Beth Israel Deaconess Medical Center, United States of America

## Abstract

**Background:**

Taxol is a microtubule stabilizing agent that arrests cells in mitosis leading to cell death. Taxol is widely used to treat breast cancer, but resistance occurs in 25–69% of patients and it is vital to understand how Taxol resistance develops to improve chemotherapy. The effects of chemotherapeutic agents are overcome by survival signals that cancer cells receive. We focused our studies on autotaxin, which is a secreted protein that increases tumor growth, aggressiveness, angiogenesis and metastasis. We discovered that autotaxin strongly antagonizes the Taxol-induced killing of breast cancer and melanoma cells by converting the abundant extra-cellular lipid, lysophosphatidylcholine, into lysophosphatidate. This lipid stimulates specific G-protein coupled receptors that activate survival signals.

**Methodology/Principal Findings:**

In this study we determined the basis of these antagonistic actions of lysophosphatidate towards Taxol-induced G2/M arrest and cell death using cultured breast cancer cells. Lysophosphatidate does not antagonize Taxol action in MCF-7 cells by increasing Taxol metabolism or its expulsion through multi-drug resistance transporters. Lysophosphatidate does not lower the percentage of cells accumulating in G2/M by decreasing exit from S-phase or selective stimulation of cell death in G2/M. Instead, LPA had an unexpected and remarkable action in enabling MCF-7 and MDA-MB-468 cells, which had been arrested in G2/M by Taxol, to normalize spindle structure and divide, thus avoiding cell death. This action involves displacement of Taxol from the tubulin polymer fraction, which based on inhibitor studies, depends on activation of LPA receptors and phosphatidylinositol 3-kinase.

**Conclusions/Significance:**

This work demonstrates a previously unknown consequence of lysophosphatidate action that explains why autotaxin and lysophosphatidate protect against Taxol-induced cell death and promote resistance to the action of this important therapeutic agent.

## Introduction

Breast cancer is the most common malignancy among women in Western societies and approximately 30% of breast cancer patients develop metastases and die [1]. Taxol is widely used for treating metastatic and early-stage breast cancer. Taxol interacts with β-tubulin [2] causing lateral polymerization and microtubule stability resulting in mitotic arrest and cell death [3]. Resistance to Taxol is common with response rates of only 25 to 69% when used as a first-line treatment. There is an urgent need to identify patients who will respond to treatment [4] and to understand how to overcome chemo-resistance.

The efficacy of chemotherapy is often compromised by survival signals received by tumor cells [5], [6]. We showed that extracellular lysophosphatidate (LPA) provides such a survival signal. LPA strongly antagonizes Taxol-induced death in MCF-7 breast cancer and MDA-MB-435 melanoma cells [7]. This effect requires the activation of phosphatidylinositol 3-kinase (PI3K) and it is accompanied by a reversal of the Taxol-induced increase in ceramide concentrations. These latter results are compatible with earlier studies where ceramides were shown to antagonize the stimulation of cell division by LPA [8]. Ceramides are bioactive lipids that cause increased apoptosis in most cells [9]. They accumulate in cancer cells in response to a large variety of chemotherapeutic agents and radiation therapy as part of the process leading to caspase activation and cell death [10], [11], [12]. Therefore, a combination of ceramides with traditional chemotherapy drugs may have the potential to be used as a new therapeutic intervention against multiple cancers [13].

The present studies deal mainly with another novel effect of LPA, namely its ability to antagonize the Taxol-induced accumulation of cancer cells in the G2/M phase of the cell cycle [7]. The signaling effects of extracellular LPA are mediated by at least eight G-protein coupled receptors [14], [15], [16]. Most of the LPA in extracellular fluids is produced by the secreted enzyme, autotaxin (ATX), which converts the abundant extracellular lysophosphatidylcholine to LPA and thus controls LPA concentrations [6], [16], [17], [18]. Circulating LPA is turned over rapidly with half-life about 3 min in mice [19], [20]. This half life depends on the balance of ATX activity in producing LPA [19] and the ecto-activities of lipid phosphate phosphatases (LPPs), which degrade extracellular LPA [6], [20], [21].

Increased ATX expression is strongly associated with tumor growth, invasion, angiogenesis and metastasis [22], [23], [24]. Recent work *in vivo* supports the importance of ATX and LPA in tumor development. Increased expression of ATX, LPA_1_, LPA_2_ or LPA_3_ receptors in mice increased the frequency of invasive, estrogen receptor-positive and metastatic breast cancer [25]. ATX activity is required for lysophosphatidylcholine to stimulate cancer cell migration [19], [26], [27] and to antagonize Taxol-induced cell death [7]. ATX action also antagonizes carboplatin-induced apoptosis in ovarian cancer cells [28].

We proposed that inhibiting ATX activity or expression, and thereby LPA formation, could provide an important supplement for chemotherapy or surgery [7], [26]. The present work was performed to identify how LPA production by ATX decreases the Taxol-induced accumulation of cells in G2/M, an event that precedes apoptosis [7]. This work is a necessary initial step in elucidating the signaling pathways used by LPA to cause Taxol resistance. We now show that this LPA action does not depend on increased expulsion of Taxol from cancer cells, increased Taxol metabolism, a delay in the entry into G2/M or selective killing of cells in G2/M. Surprisingly, LPA has a remarkable effect of enabling cells that had been arrested in G2/M by Taxol to normalize spindle formation, divide and thus escape from cell death. This LPA action depends on the activation of PI 3-kinase, which causes Taxol to be displaced from the tubulin polymer fraction.

## Results

LPA decreases the Taxol-induced accumulation of cells in G2/M by increasing the escape from mitotic arrest.

**Figure 1:**
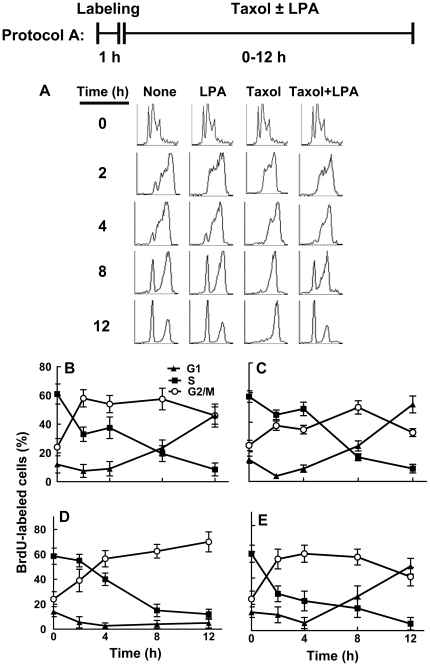
Lysophosphatidate does not block the entry of Taxol treated MCF-7 cells into G2/M. Panels A–E, cells were treated with BrdU for 1 h to label cells in S-phase. Cells were then incubated for 12 h with 10% delipidated serum in the presence or absence of 50 nM Taxol or 5 µM LPA (Protocol A). The progression of cells through S, G2/M and G1 phases was quantified (Panels B–E) from the FACS analysis (Panel A). DNA histograms were composed with CellQuest software. The histogram for time 0 for all treatments shows the same results, repeated for clarity of the figure. Results are means ± SD for three independent experiments.

We selected MCF-7 cells for this study based on our previous work [7] and the widespread use of these cells to study apoptosis. To investigate how LPA antagonizes the Taxol-induced arrest in G2/M and subsequent cell death [7], we determined if LPA affects cell cycle progression or induces selective killing of cells arrested in mitosis by Taxol. MCF-7 cells were treated with bromodeoxyuridine (BrdU) for 1 h to label cells in S-phase (Protocol A; Fig. 1). Progression through the cell cycle of the labeled cells was measured by FACS analysis for 12 h after treatment with 10% delipidated serum, in which LPA and other bioactive lipids were removed [7] and 50 nM Taxol was added in the presence or absence of 5 µM LPA. These concentrations gave optimum cell killing and efficient rescue, respectively [7]. This Taxol concentration is similar to that achieved during chemotherapy [29] and the LPA concentration is in physiological/pathological range [17].

Sixty percent of the labeled cells were still in S-phase at the end of the labeling period, as expected. Untreated cells started to exit S-phase immediately thereafter, and approximately 60% of these labeled cells had progressed into the G2/M phase at 2 h after labeling (Fig. 1A,B). Labeled cells started to exit mitosis, and enter into the G1 phase after 4 h from labeling and they continued to accumulate in the G1 peak during 12 h after labeling. LPA alone slowed the progression of BrdU-labeled cells out of S-phase. Fewer cells accumulated in G2/M until 8 h after labeling. However, there was no significant change in the progression of BrdU-labeled cells through mitosis and into the following G1 phase (Fig. 1A,B,C). The percentage of cells in the G1 peak increased in parallel in LPA treated and control cells from 4 to 12 h after labeling.

Taxol slightly delayed the transit of labeled cells out of S-phase, resulting in a lower decrease of cells in S-phase for the first 4 h of treatment. It also inhibited progression through mitosis, as expected [30]. The percentage of labeled cells in the G2/M peak increased continuously from the time of Taxol addition up to 12 h later (up to 70±14% at 12 h), whereas there was no significant progression of labeled cells through mitosis into the following G1 (Fig. 1A,D). Addition of LPA to the Taxol treatment restored the passage of the cells through S-phase to the levels observed in the absence of Taxol. Cells treated with both Taxol and LPA started to exit S-phase immediately after labeling. Approximately 60% of labeled cells had progressed into the G2/M phase at 2 h after labeling. Labeled cells started to exit mitosis and enter into the G1 phase after 4 h from labeling. They continued accumulating in the G1 peak to 12 h after labeling, much like the untreated cells did. In fact, the percentage of cells in the G1 peak increased as fast in Taxol and LPA treated cells as it did in control cells from 4 to 12 h after labeling (Fig. 1A,E). LPA, therefore, enabled the Taxol-treated cells to start exiting G2/M after 4 h. There was consequently a marked increase in accumulation of cells in the G1 peak from 6±5% at 4 h to 49±9% after 12 h incubation (p<0.05) (Fig. 1A,E). BrdU-labeled cells, therefore, progressed through the cell cycle and accumulated in the G1 peak. This evidence indicates that LPA releases the cells from G2/M arrest in the presence of Taxol.

**Figure 2:**
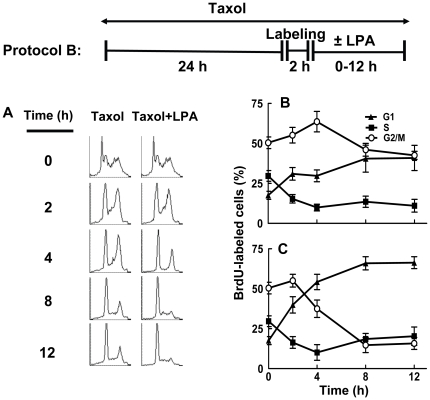
Lysophosphatidate releases the cells from G2/M phase. Cells were preincubated with 50 nM Taxol for 24 h and cells passing through S-phase were labeled for 2 h with BrdU. Cells were then incubated for 12 h with 10% delipidated serum in the presence or absence of 50 nM Taxol or 5 µM LPA (Protocol B). The progression of cells treated with Taxol alone or Taxol and LPA through S, G2/M and G1 phases of the cell cycle (Panels B and C, respectively) was quantified by FACS analysis (Panel A). DNA histograms were composed with CellQuest software. The histogram for time 0 for all treatments shows the same results, repeated for clarity of the figure. Results are means ± SD for three independent experiments.

To investigate further this apparent progression through mitosis in the presence of Taxol and LPA, we pretreated MCF-7 cells with 50 nM Taxol in the presence of 10% delipidated serum for 24 h to establish a G2/M arrest. We then labeled cells that were still cycling through S-phase with BrdU for 2 h. The cells were then incubated further with Taxol in the presence or absence of LPA (Protocol B; Fig. 2). Maintaining Taxol increased (p<0.05) the percentage of BrdU-labeled cells in the G2/M peak from 50±8% at the end of the labeling period to a maximum of 63±8% at 4 h later, as expected **(** Fig. 2A,B **)**. Although the mitotic block was not absolute and the percentage of labeled cells in the G2/M peak decreased after 4 h; only 40%±8% of labeled cells had re-entered into the G1 peak at 12 h after the labeling. Moreover, these cells did not progress significantly into the following S-phase. The percentage of labeled cells detected in the S-phase peak decreased during the first 4 h and remained at this low level to the end of the experiment at 12 h.

Addition of LPA to the Taxol-treated cells decreased (p<0.05) the percentage of labeled cells in the G2/M peak from 4 h until 12 h after time of addition (Fig. 2A,C). After 12 h only 19±5% remained in G2/M. Consistent with a release from the G2/M block, LPA consistently induced the expected concomitant increase in the percentage of labeled cells detected in the G1 peak from 17±4% at the time of addition to 68±7% at 12 h later (p<0.01) (Fig. 2A,B,C). These results show that LPA does not produce a decrease in the percentage of cells in G2/M by inducing selective killing of these cells. Instead, it induces their release from the Taxol-induced G2/M arrest. The cells released from mitotic arrest underwent mitosis and started another cell cycle, entering the following G1 phase in less than 4 h, and the following S phase after 8 h (Fig. 2A,C).

**Figure 3:**
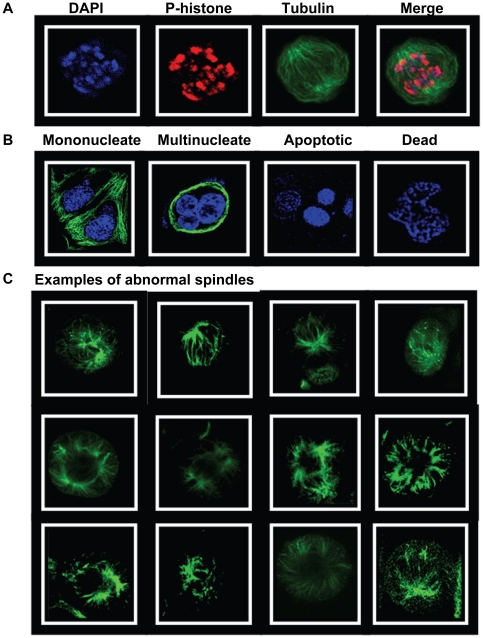
Morphology of Taxol-treated MCF-7 cells. MCF-7 cells were preincubated with 10% delipidated serum and 50 nM Taxol for 26 h. Cells were then stained with DAPI, anti-phospho-histone and with anti-tubulin. The appearance of the cell in mitosis (assessed by DAPI and P-histone staining) is shown in Panel A. Panel B shows cells that did not stain for P-histone and which were identified as being mononucleated, multinucleated or dead. Panel C shows examples of the appearance of cells that were identified as having abnormal spindles.

We next verified these highly unexpected conclusions by studying the effects of Taxol and LPA on cell death and the morphology of the nucleus and spindles in MCF-7 compared to MDA-MB-468 cells. MCF-7 cells do not express caspase-3 [31] whereas MDA-MB-468 cells express relatively high caspase-3 activity [32]. We incubated both cell lines for 26 h with 10% delipidated serum and 50 nM Taxol and then used microscopy to study their fates in the presence or absence of LPA (Protocol C). We also removed Taxol from the incubations for the last 12 h to test if cells recovered from Taxol treatment without LPA addition. Cells were stained with anti-tubulin antibody, anti-phospho-histone H3 antibody and DAPI for DNA (Fig. 3A). This identified mononucleated cells, multinucleated cells, those that were in apoptosis or dying (obvious deformations in cytoskeletal and nuclear structure) (Fig. 3B) and those in mitosis with abnormal spindle morphology (Fig. 3C).

**Figure 4:**
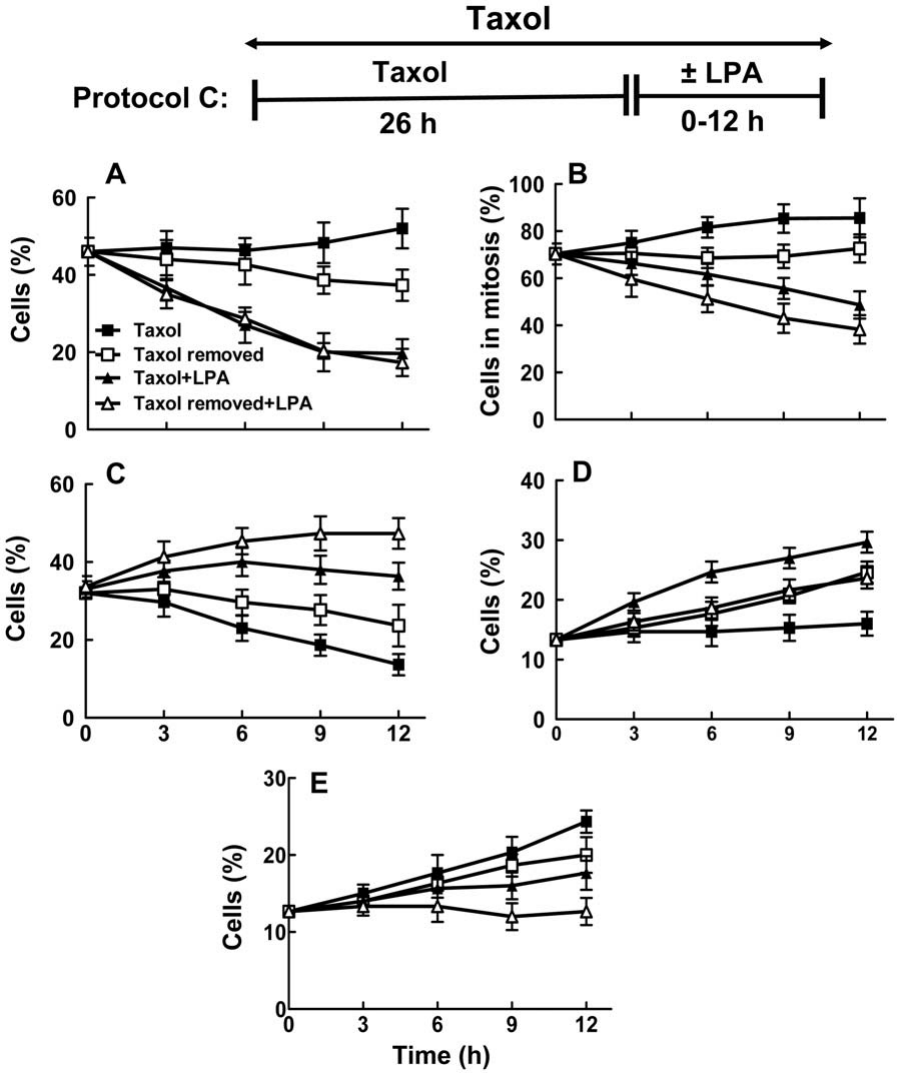
Lysophosphatidate releases MCF-7 cells from Taxol-induced arrest in G2/M and cell death. MCF-7 cells were preincubated with 10% delipidated serum and 50 nM Taxol for 26 h. Taxol was then maintained or removed in the presence of 10% delipidated serum and 5 µM LPA was added as indicated (Protocol C). Cells were then stained with DAPI, anti-phospho-histone and with anti-tubulin at the times indicated. The Panels show the percentage of: A) cells in mitosis, B) percentage of cells in mitosis that had abnormal spindles, C) mononucleated cells, D) multinucleated cells and E) dead and dying cells. Total number of cells (100%) includes mononucleated, multinucleated, dead and dying and mitotic cells. Time zero shows 26 h pretreatment with Taxol. Results are means ± SD (where large enough to be shown) for three independent experiments.

After incubating with Taxol for 26 h, 46% of MCF-7 cells were in mitosis (Fig. 4A). Incubating for a further 12 h in Taxol increased (p<0.05) the percentage of cells in mitosis to 52±10%, whereas removal of Taxol decreased this value to 40±5%, p<0.05. Incubating cells with LPA in the presence or absence of Taxol for 12 h decreased (p<0.05) the percentage of cells that were in mitosis to about 20% (Fig. 4A). LPA also decreased (p<0.05) the proportion of mitotic cells with abnormal spindles from 86±9% in cells where Taxol was maintained to 49±7% (Fig. 4B). Removal of Taxol decreased the percentage of the cells with abnormal spindles and the presence of LPA further amplified this effect (p<0.05). LPA also facilitated mitosis (p<0.05) and the production of G1 mononucleated cells regardless of whether Taxol was maintained or removed (Fig. 4C). In addition, LPA increased the percentage of multinucleated cells (Fig. 4D). This latter result indicates that some cells escaped Taxol-induced mitotic arrest in the absence of cytokinesis.

After incubating with Taxol for 26 h, 13±3% of the remaining cells were dead or dying as identified because of their apoptotic or necrotic morphology (Fig. 3B). Incubating the cells for a further 12 h in Taxol increased (p<0.05) the percentage of dead or dying cells to 24±4% (p<0.01) and this value was similar when Taxol was removed. LPA decreased (p<0.05) the percentage of dead or dying cells regardless of whether Taxol was maintained or removed during the final 12 h of incubation (Fig. 4E).

**Figure 5:**
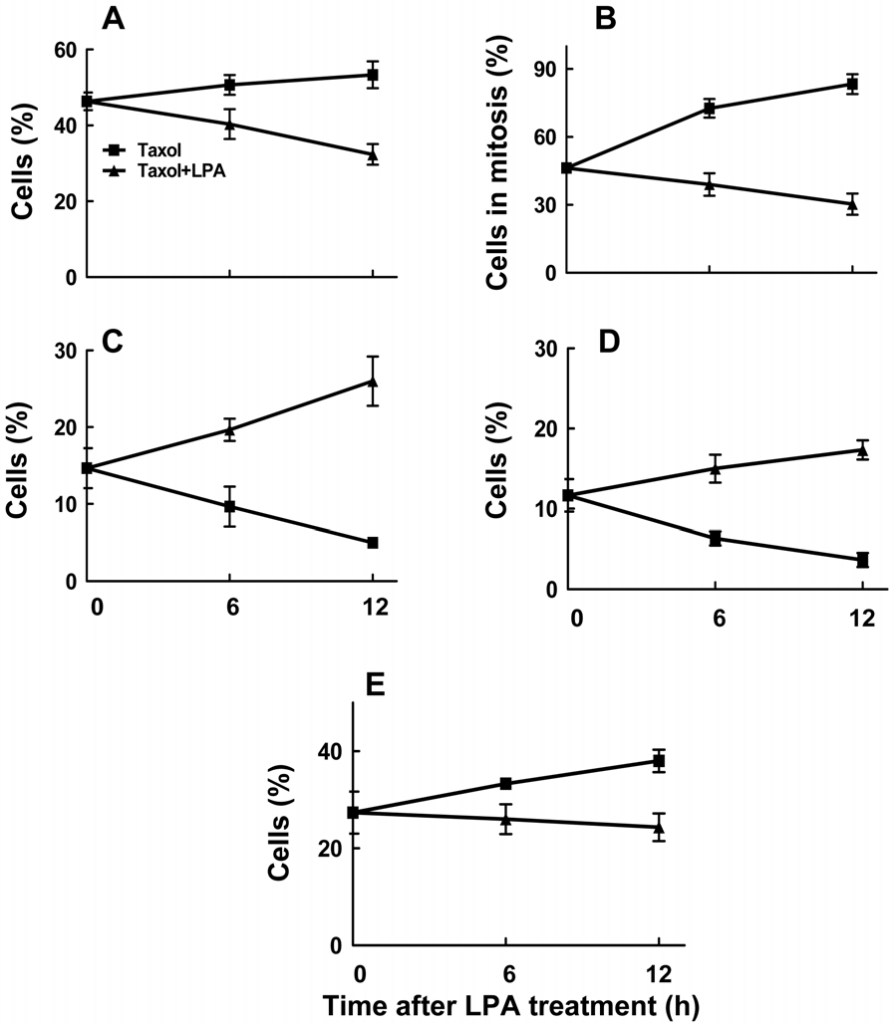
Lysophosphatidate releases MDA-MB-468 cells from Taxol-induced arrest in G2/M and cell death. MDA-MB-468 cells were preincubated with 10% delipidated serum and 50 nM Taxol for 26 h. Taxol was then maintained or removed in the presence of 10% delipidated serum and 5 µM LPA was added as indicated (Protocol C). Cells were stained with DAPI, anti-phospho-histone and with anti-tubulin at the times indicated. The Panels show the percentage of: A) cells in mitosis, B) percentage of cells in mitosis that had abnormal spindles, C) mononucleated cells, D) multinucleated cells and E) dead and dying cells. Total number of cells (100%) includes mononucleated, multinucleated, dead and dying and mitotic cells. Time zero shows 26 h pretreatment with Taxol. Results are means ± SD for three independent experiments.

We also used MDA-MB-468 cells to establish if the effects of LPA on Taxol-induced mitotic arrest and cell death occur in other breast cancer cells. Treatment for 26 h with 50 nM Taxol caused about 46±5% to accumulate in mitosis (Fig. 5A). We then chose to incubated these cells for 6 or 12 h with LPA since this was where the major effects of LPA were observed in MCF-7 cells (Fig. 4). Incubating MDA-MB-468 cells with LPA in the presence of Taxol for 12 h decreased (p<0.05) the percentage of cells that were in mitosis from 53±6% with Taxol alone to about 32±5% (Fig. 5A). The percentage of cells with abnormal spindles increased with time of incubation, whereas LPA reversed this effect (p<0.05) (Fig. 5B). LPA also increased (p<0.05) the percentage of mononucleated cells in G1 from 14±4% to 20±3% and 26±6% after 6 and 12 h, respectively (Fig. 5C). LPA also significantly increased the percentage of multinucleated cells after 6 and 12 h (Fig. 5D). By contrast, continued treatment with Taxol in the absence of LPA decreased the proportion of mononucleated and multinucleated cells. Incubation with Taxol for the initial 26 h of the experiment induced significantly higher killing of MDA-MB-468 cells compared to MCF-7 cells (27±7% compared to 13±3%) (Figs 4E and 5E). An additional incubation of MDA-MB-468 cells for 12 h with Taxol increased the percentage of dead and dying cells to 38±4%. LPA prevented the increase in the percentage of dead and dying cells and this percentage stayed at 24±5% after the 12 h incubation.

LPA does not increase the expulsion of Taxol from MCF-7 cells or increased Taxol metabolism, but it does cause dissociation of Taxol from the tubulin polymer compartment.

The next experiments were performed to investigate the mechanisms of how LPA decreases Taxol-induced cell death through the release of breast cancer cells from mitotic arrest. Chemo-resistance is already known to result partially through the activation of drug transporters [33], which can cause cross-resistance to other chemotherapeutic agents [34]. We, therefore, hypothesized that LPA decreases Taxol-induced accumulation of the cells in G2/M [7] by stimulating Taxol efflux through ABCB1 (multidrug resistance gene 1), *p*-glycoprotein (ABCC1) or ABCG2, since these transporters account for a major portion of drug resistance in human tumor cells [35]. MCF-7 cells express high levels of ABCG2, less ABCC1 and no ABCB1 [36].

**Figure 6:**
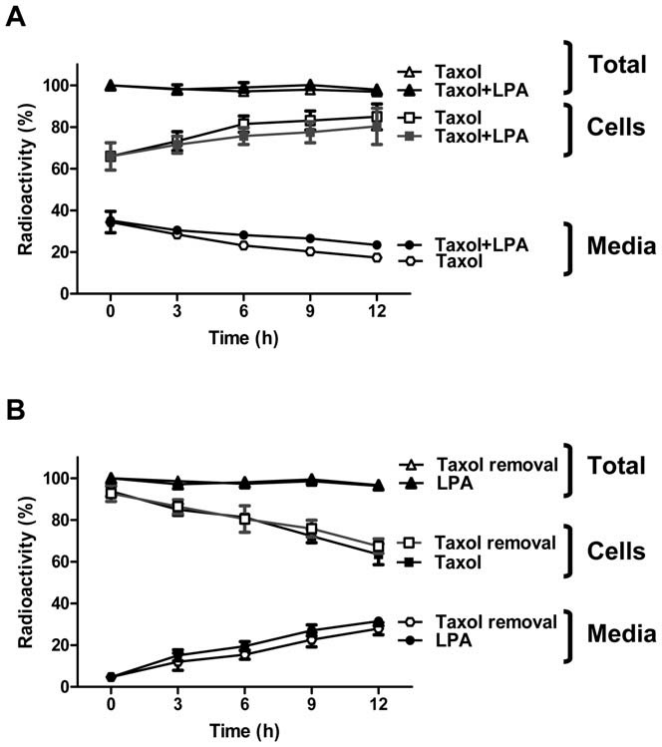
LPA does not affect Taxol expulsion form MCF-7 cells. Panel A, approximately 2×10^5^ MCF-7 breast cancer cells were grown to confluence in 3.5 cm cell culture dishes. The medium was replaced with 1.5 ml RPMI 1640 medium containing 10% of charcoal-treated FBS with 50 nM Taxol containing 0.5 µCi [^3^H]Taxol. After incubation for 26 h, the medium was supplemented in some cases with 5 µM LPA or not as indicated and the incubation was continued for another 12 h. Media and cells were collected at the times indicated and Taxol was extracted. Results are expressed as the percentage of total radioactivity recovered in the cells and medium. Panel B, cells were treated and analyzed as above but after 26 h incubation, Taxol was removed and 5 µM LPA was added or not as indicated. The results are expressed as the percentage of the radioactivity added to the medium presented as mean ± S.D. (where large enough to be shown) for three independent experiments.

To investigate whether LPA stimulates the export of Taxol, MCF-7 cells were pretreated for 26 h with 50 nM [^3^H]Taxol to load the cells with Taxol as in the experiments described in Fig. 4. These cells were incubated in 10% delipidated serum, in which endogenous LPA and other bioactive lipids were removed [7]. Cells were then incubated in the presence or absence of 5 µM LPA for a further 12 h. At the end of the 26 h incubation, 70±8% of the total Taxol was in the cells and incubation for a further 12 h increased (p<0.05) the proportion of Taxol inside the cells to 80±5% (Fig. 6A). Removal of Taxol during the final 12 h incubation was accompanied by the loss (p<0.05) of 32±8% of the [^3^H]Taxol from the cells into the medium (Fig. 6B). LPA did not significantly affect these Taxol distributions in either protocol. We, therefore, rejected our first hypothesis.

We also analyzed the [^3^H]Taxol under all conditions by thin layer chromatography [37]. There was no significant formation of 3′-*p*-hydroxytaxol and 6α-hydroxytaxol, the major Taxol metabolites, either in the absence or presence of LPA (results not shown).

**Figure 7:**
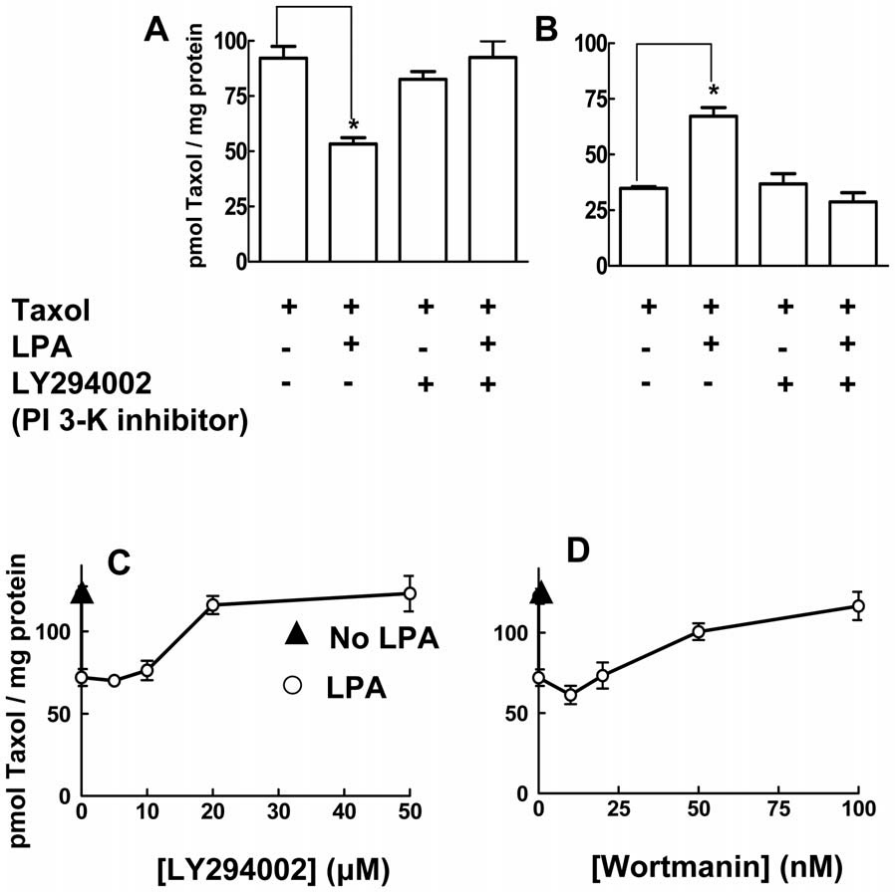
LPA causes dissociation Taxol from the polymerized tubulin compartment. Approximately 2×10^6^ cells were seeded in 6 cm dishes. After growing cells to confluence, the medium was replaced with 3 ml RPMI 1640 containing 10% of charcoal-treated FBS containing 50 nM [^3^H]Taxol (0.5 µCi). Cells were preincubated for 26 h. After this, 5 µM LPA and different concentrations of inhibitors were added as indicated. Polymerized tubulin (Panel A) and soluble fractions (Panel B) were then separated and the distribution of [^3^H]Taxol was determined. Panels C and D show the dose response curves for LY294002 and Wortmannin, respectively. Results are means ± SD for three independent experiments. Panels A and B, * a significant difference of *P<0.05.*

We also determined whether the LPA-induced escape of the breast cancer cells from mitotic arrest can be explained by the release of Taxol from its association with the polymerized tubulin fraction. MCF-7 cells were pretreated for 26 h with 50 nM [^3^H]Taxol to load the cells with Taxol and produce mitotic arrest as in Fig. 4. The medium was maintained, 5 µM LPA was added as indicated and the cells were then incubated for and additional 12 h. The amount of [^3^H]Taxol associated with the polymerized tubulin fraction was decreased significantly by LPA treatment (Fig. 7A). This decrease was balanced by the appearance of Taxol in the soluble compartment of the cells (Fig. 7B) in agreement with the lack effect of LPA on the cellular content of Taxol (Fig. 6A and B).

The LPA effect in decreasing the association of Taxol with the polymerized tubulin was reversed by incubation with 40 µM LY294002, the PI 3-kinase inhibitor (Fig. 7A). We confirmed these results in separate experiments where MCF-7 cells were incubated in the presence or absence of LPA or LY294002 for 6 rather than 12 h (results not shown). We were aware that LY294002 has other actions such as the inhibition of casein kinase-2. We, therefore, tested different concentrations of LY294002, Wortmannin (another PI3K inhibitor) and TBB (4,5,6,7-tetrabromobenzotriazole, a selective casein kinase-2 inhibitor) [38]. LY294002 reversed the LPA-induced displacement of Taxol from polymerized tubulin with a maximum effect at 20 µM (Fig. 7C). Wortmannin also had a similar effect at 50 and 100 nM (Fig. 7D). TBB had no significant effect between 10 and 100 µM (results not shown).

We next investigated the role of LPA receptors on the LPA effect on Taxol distribution.

**Figure 8:**
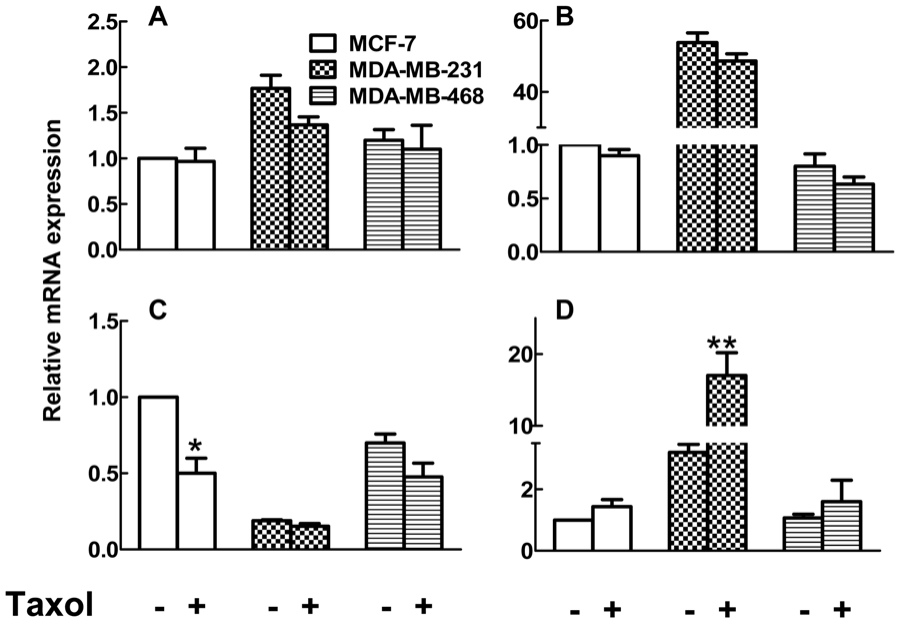
Differential expression of autotaxin and LPA_1-3_ receptors in MCF-7, MDA-MB-231 and MDA-MB-468 breast cancer cells. Results show mRNA concentrations expressed relative to that for cyclophilin for (A) ATX (B) LPA_1_ (C) LPA_2_ and (D) LPA_3_ in cells incubated for 26 h in the presence or absence of Taxol. Results are means ± SD from three independent experiments for ATX and LPA_1_ and six independent experiments for LPA_2_ and LPA_3_. ^*^ *P*<0.05 and ** p<0.01.

Among the LPA receptors, LPA_1_, LPA_2_, and LPA_3_ are the best characterized and have the major role in LPA action [6], [39]. Real-time RT-PCR was performed to detect changes in mRNA expression levels for LPA receptors and also ATX in three breast cancer cell lines. We already showed that ATX expression is low in MCF-7 and MDA-MB-231 cells (Samadi et al; Gaetano et al) and ATX mRNA levels are similar in the MDA-MB-468 cells (Fig. 8A). Taxol treatment for 26 h did not significantly change ATX mRNA expression in any of the cell lines.

MCF-7 and MDA-MB-468 cells predominantly express LPA_2_ and MDA-MB-231 mainly express LPA_1_ receptors [40] and this is reflected in the mRNA levels (Fig. 8B and C). MDA-MB-231 cells expressed relatively more mRNA for LPA_3_ than MCF-7 or MDA-MB-468 cells (Fig. 8D). Taxol treatment caused a 6-fold increase in LPA_3_ receptor mRNA in MDA-MB-231 cells and a significant decrease in LPA_2_ mRNA in MCF-7 cells (Fig. 8B,C,D).

**Figure 9:**
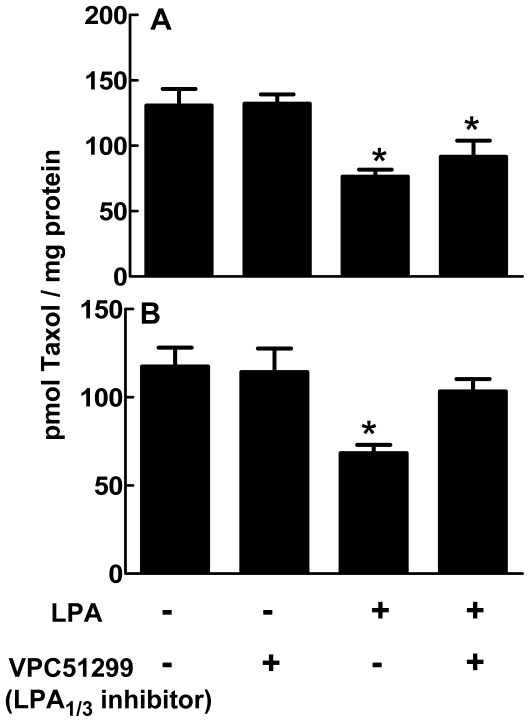
Involvement of LPA_1/3_ and LPA_2_ receptors in LPA-induced displacement of Taxol from polymerized tubulin in MCF-7 and MDA-MB-231 cells. MCF-7 (Panel A) and MDA-MB-231 cells (Panel B) were preincubated for 26 h with [^3^H]Taxol as in Fig. 7. After this, 5 µM LPA or 1 µM VPC51299, a LPA_1/3_ receptor antagonist, was added as indicated and the incubations were continued for a further 12 h. Polymerized tubulin was then isolated and the distribution of [^3^H]Taxol was determined. Results are means ± SD for three independent experiments. *P<0.05 comparing cells incubated in the absence of LPA with those incubated with LPA and/or the LPA_1/3_ antagonist, VPC51299.

To investigate the role of these LPA receptors in the displacement of Taxol from polymerized tubulin, we applied VPC51299, an LPA_1/3_, antagonist and studied Taxol localization in both cytosolic and polymerized tubulin compartments. In MCF-7 cells, 1 µM VPC51299 failed to change the LPA-induced displacement Taxol from polymerized tubulin (Fig 9A). However, in MDA-MB-231 cells, VPC51299 attenuated the action of LPA (Fig 9B).

## Discussion

The present work demonstrates that the effect of LPA in decreasing the Taxol-induced accumulation of breast cancer cells in G2/M [7] does not depend on an LPA-induced export of Taxol from the cells, or increased Taxol metabolism. Neither does LPA decrease the Taxol-induced accumulation of MCF-7 cells in mitosis by delaying entry into G2/M or by inducing selective death of the cells arrested in mitosis. Instead, LPA produced the surprising effect of reversing Taxol action in producing abnormal mitotic spindles. This effect was dependent on PI3K and the displacement of Taxol from its association with the tubulin polymer fraction. We also showed that the LPA-induced release from mitotic arrest protected the cells from the consequent Taxol-induced cell death [41], [42], [43].

We designed our experiments to investigate this latter phenomenon by preincubating cells for 26 h with delipidated serum and Taxol to produce mitotic arrest. The delipidated serum provides protein growth factors required for cell cycle progression, but it lacks lipid growth factors including LPA [7]. This enabled us to study the effects of Taxol removal or LPA addition on the structure of spindles in the cells and the progression of cells from G2/M into G1. Although removal of Taxol after 26 h caused a decrease in Taxol within the cells and allowed more cells to progress to G1, this effect was not as potent as the addition of LPA. LPA did not increase the expulsion of Taxol from the cells, but it did cause a redistribution of Taxol within the cells by displacing it from the polymerized tubulin fraction. We, therefore, conclude that LPA has a specific effect in facilitating normal spindle structure in the presence of Taxol. This LPA effect will decrease Taxol-induced mitotic arrest and also enable cancer cells to escape from Taxol-induced arrest in G2/M and cell death, as we showed using our experimental protocols.

*In vivo,* ATX expression increases the exposure of tumors to LPA and this is associated with increased tumor aggressiveness, angiogenesis and metastasis [22], [23], [24], [44]. MCF-7 cells express mainly LPA_2_ [40] and, as expected, neither LPA_1_ and LPA_3_ antagonists or pertussis toxin blocked LPA antagonism towards Taxol in these cells [7]. LPA also enabled MDA-MB-468 cells to escape from Taxol-induced mitotic arrest and consequent cell death. These cells also have high LPA_2_ receptor expression [40], but whereas MCF-7 cells do not express caspase-3 [31], MDA-MB-468 cells express relatively high caspase-3 activity [32]. This probably explains why they displayed greater Taxol-induced cell death. LPA_2_ receptors are especially associated with increased development of breast and other cancers [25], [45]. Activation of LPA_2_ receptors protects against adriamycin-induced caspase-3 cleavage and cell death [46]. This depends on the LPA_2_-dependent degradation of the pro-apoptotic protein, Siva-1 [46].

Our work describes another mechanism by which LPA can protect against Taxol-induced cell death, which involves displacement of Taxol from the polymerized tubulin fraction and escape from mitotic arrest. We also showed that LPA protects MDA-MB-435 cells, which show relatively high LPA_1_ expression, from Taxol-induced death [40]. Consequently, the effects of LPA in causing Taxol resistance appear to occur in several cancer cell lines. We showed that LPA-induced PI3K activation decreases the Taxol-induced formation of ceramide, a proapoptotic lipid [7]. Now we showed that the role of LPA in displacement of Taxol from polymerized tubulin depends on the differential expression of LPA receptors (LPA _1/3_ in MDA-MB-231 cells and LPA_2_ in MCF-7 cells). In fact, Taxol treatment increases mRNA expression for LPA_3_ in MDA-MB-231 cells and decreased LPA_2_ mRNA expression in MCF-7 cells. We do not know the mechanisms by which Taxol effects these changes.

Activation of several LPA receptors, including LPA_1_, LPA_2_ and LPA_3_ results in PI3K activation [47]. We propose that LPA-induced displacement of Taxol from polymerized tubulin is PI3K dependent based on studies with LY294002 and wortmannin where we see inhibition in the expected concentration ranges. We recognize that all inhibitors have off target effects, but these are different between LY294002 and wortmannin. Also, we used a casein kinase-2 inhibitor since LY294002 can inhibit this enzyme [38]. Casein kinase-2 can be activated in Wnt signaling, and it is also involved in cell cycle progression [48], [49]. To investigate the possible role of casein kinase-2 in the LPA effect, we also used TBB (a selective inhibitor of casein kinase-2) and observed no effect on LPA-induced Taxol displacement from polymerized tubulin even at 100 µM TBB.

Tumor cells are particularly responsive to the effects of ATX and LPA compared to non-transformed cells since they often express low LPP activities [6], [50], [51], [52], [53]. In addition, MCF-7 and MDA-MB-435 cells exhibit very low expression of LPP1 and LPP3, and relatively high LPP2 compared non-transformed MCF10A breast epithelial cells (G. Venkatraman and D.N. Brindley, unpublished observations based on real-time RT-PCR). This distribution is opposite to that in non-transformed fibroblasts [54], [55]. The LPPs have a dual function: the ecto-activities balance the action of ATX by decreasing extracellular LPA concentrations and thus receptor activation [20], [21]. Increased LPP1 expression enhances LPA degradation by ovarian cancer cells, which inhibits cell proliferation and colony-forming activity, and produces a marked increase in apoptosis [52]. The intracellular activities of the LPPs decrease signaling downstream of the activation of LPA and tyrosine kinase receptors including the platelet-derived growth factor receptor [54], [56]. Importantly, high LPP1 expression also decreases the ability of LPA to activate downstream signaling targets including ERK, Rac and Rho [54]. This appears to depend upon the expression of LPP1 within the cell and its ability to degrade a wide variety of bioactive lipid phosphates downstream of receptor activation [56]. Increased expression of LPP3 also decreases the growth, survival, and tumorigenesis of ovarian cancer cells [53]. By contrast, LPP2 has effects on cell signaling that are different from those of LPP1 and LPP3 since LPP2 expression specifically stimulates the progression of cells through the cell cycle [55]. We, therefore, conclude that many cancer cells may be particularly sensitive to the effects of LPA [6]. The LPA effects that we observe on mitosis in MCF-7 cells could, therefore, be higher than those in non-transformed cells.

The present work establishes that the effects of ATX and LPA in antagonizing Taxol-induced death of breast cancer cells results from the PI3K-dependent displacement of Taxol from polymerized tubulin and escape from mitotic arrest. This is an unexpected observation that adds to our knowledge of how LPA signaling downstream of PI3K antagonizes Taxol action. Our work also provides further evidence that ATX and LPA could be an important source of chemo-resistance to the therapeutic use of Taxol in treating breast and other cancers. The roles of ATX and LPA in aggravating resistance to chemotherapy have received little attention. No present cancer treatment depends on the inhibition of ATX or LPA signaling. This work emphasizes the importance of developing therapeutic agents that decrease ATX activity and signaling through LPA receptors. These agents could provide valuable adjuvants to increase the efficacy chemotherapy and surgery.

## Materials and Methods

The sources of most of the materials and details of methods including the preparation of delipidated calf serum have been described [7].

### Cells

Characterized MCF-7, MDA-MB-231 and MDA-MB-468 cells were obtained from the American Type Culture Collection (Manassas, VA) and they were used within six months after resuscitation.

### Materials

Monoclonal anti-α-tubulin, sodium pyruvate, NADH, RNAase A, goat and donkey serum, sodium borate, and propidium iodide were from Sigma-Aldrich (Oakville, ON, Canada). Bromodeoxyuridine (BrdU), mouse anti-BrdU and FITC goat anti**–**mouse IgG antibody were purchased from BD Pharmingen (Mississauga, ON, Canada). [*o-*benzamido-^3^H]Taxol was obtained from Moravek Biochemicals (Brea, CA, USA). Anti-phospho-histone H3 was from Millipore (Billerica, MA, USA). We purchased TBB (casein kinase-2 inhibitor) from Tocris Biosciences (Ellisville, MI, USA).

### Cell culture and bromodeoxyuridine/propidium iodine double staining

Bromodeoxyuridine/propidium iodine double staining was performed as described previously [57]. For nuclei isolation, we optimized incubation times and temperatures for enzymatic digestion in MCF-7 cells (5 ml of 0.04% pepsin in 0.1 N HCl for 20 min at room temperature). Samples were analyzed with a Calibur Flow Cytometer (Becton Dickinson, San José, CA, USA) using Cell Quest software.

### Immunocytochemistry

Flame-sterilized glass coverslips (18 mm) were placed in 12-well culture plates and about 2×10^5^ MCF-7 breast cancer cells were plated and grown to confluence. Cells were stained with DAPI, monoclonal anti-α-tubulin and polyclonal anti-histone H3.1 (Phospho-Ser 10) [58].

### Measurement of Taxol uptake, distribution and metabolism

After growing cells to confluence, the medium was replaced with 1.5 ml RPMI 1640 containing 10% of charcoal-treated FBS containing 50 nM [^3^H]Taxol (6,660 Ci/mol). Cells were incubated for the indicated times and medium was collected. Cells were washed three times with ice-cold RPMI 1640 medium containing 0.1% BSA to remove extracellular Taxol. The cell lysates were then collected by scrapping twice with 0.5 ml of ice-cold methanol. Chloroform (1 ml) was added to the combined methanol extracts followed by 0.9 ml of 2 M KCl. The mixture was shaken and centrifuged. Radioactivity in both cell lysates and media were measured by scintillation counting. Cell extracts were also analyzed on silica gel 60 thin layer chromatography plates (Merck, Darmstadt, Germany) after development with toluene/acetone/formic acid (60∶39∶1, by vol.) [37]. The distribution of ^3^H was determined with a BioScan 200 Imaging Scanner followed by scintillation counting of various areas of the plate.

### Measurement of Taxol association with polymerized tubulin

Incorporation of Taxol in the soluble (cytosolic) and polymerized (non-cytosolic) fractions of the cells was determined by a standard method as described previously [59], [60]. Briefly, 2×10^6^ cells were seeded in 6 cm dishes. After growing cells to confluence, the medium was replaced with 3 ml RPMI 1640 containing 10% of charcoal-treated FBS and 50 nM [^3^H]Taxol (0.5 µCi). Cells were incubated for 26 h. Then the incubation was continued for additional 12 h with [^3^H]Taxol in the presence and absence of LPA (5 µM) and different inhibitors as described in the text. Cells were then collected and suspended in 200 µl of hypotonic lysis buffer (1 mM MgCl_2_, 2 mM EGTA, 0.5% NP-40, 20 mM Tris-HCl at pH 6.8 containing protease and phosphatases inhibitors) and incubated for 5 min. Polymerized tubulin was collected in a pellet by microcentrifugation at 18000 x g for 10 min. The supernatant (200 µl) containing the cytosolic fraction was transferred to another tube. The pellet containing the polymerized fraction was resuspended in 200 µl of hypotonic lysis buffer. Protein concentrations for each fraction were measured using the BCA assay (Thermo Scientific). A sample of each fraction (20 µl) was added to 2 ml of scintillation solution (Cyto-Scint, MP, Irvine, CA), and radioactivity counted.

### Measurement of mRNA expression

Total RNA was extracted using the RNAqueous kit, according the manufacture's instruction. DNA-free kit was also applied to remove contaminating DNA from RNA preparation. Total RNA was treated with superscript II reverse transcriptase. Real-time RT-PCR was performed with 25 µl of master mix containing 2×Syber Green buffer mix and forward and reverse primers (Invitrogen). The internal control was the constitutively expressed housekeeping human cyclophilin A. Primers for human ATXwere as follows: sense, 5′-ACAACGAGGAGAGCTG CAAT-3′; antisense, 5′-AGAAGTCCAGGCTGGTGAGA-3′ ; for human cyclophilin A: sense, 5′-TTCATCTGCACTGCCAAGAC-3′; antisense, 5′-TCGAGTTGTCCACAGT CACC-3′; for human LPA_1_: sense, 5′-ACAGCCATGAAT GAAC CA CA-3′; antisense, 5′-TCTCCGAGTATTGGGTCCTG-3′; for human LPA_2_: sense, 5′-GTGCAGGAATCTGGCTCTTC-3′; antisense, 5′-GGGTGTCCACAGTCTGTCCT-3′; for human LPA3 receptor: sense, 5′-TGCTCATTTTGCTTGTCTGG-3′ and antisense, ATGATGAGGAAGGCCATGAG. Samples were assayed in triplicate on the 7500 Real Time PCR System (Applied Biosystems).

### Statistical Analysis

Statistical analysis was performed by analysis of variance with a Kruskal-Wallis *post hoc* test.
